# Patient-derived xenografts of different grade gliomas retain the heterogeneous histological and genetic features of human gliomas

**DOI:** 10.1186/s12935-019-1086-5

**Published:** 2020-01-03

**Authors:** Wenxin Zeng, Zhaohua Tang, Yongguo Li, Guangnian Yin, Zili Liu, Jie Gao, Yan Chen, Feilan Chen

**Affiliations:** 1 Laboratory Animal Center, Chongqing Medical University, Yixueyuan Road 1, Yuzhong District, Chongqing, 400016 People’s Republic of China; 2 Neurosurgery Department, The First Affiliated Hospital of Chongqing Medical University, Chongqing, People’s Republic of China; 3 Forensic Medicine Department, Chongqing Medical University, Chongqing, People’s Republic of China; 4 Pharmaceutical College, Chongqing Medical and Pharmaceutical College, Chongqing, People’s Republic of China

**Keywords:** Patient-derived xenografts (PDXs), Gliomas, Heterogeneity, Histology, Genetics

## Abstract

**Background:**

Gliomas account for the major part of primary brain tumors. Based on their histology and molecular alternations, adult gliomas have been classified into four grades, each with distinct biology and outcome. Previous studies have focused on cell-line-based models and patient-derived xenografts (PDXs) from patient-derived glioma cultures for grade IV glioblastoma. However, the PDX of lower grade diffuse gliomas, particularly those harboring the endogenous IDH mutation, are scarce due to the difficulty growing glioma cells in vitro and in vivo. The purpose of this study was to develop a panel of patient-derived subcutaneous xenografts of different grade gliomas that represented the heterogeneous histopathologic and genetic features of human gliomas.

**Methods:**

Tumor pieces from surgical specimens were subcutaneously implanted into flanks of NOD-Prkdc^scid^ ll2rg^null^ mice. Then, we analyzed the association between the success rate of implantation with clinical parameters using the Chi square test and resemblance to the patient’s original tumor using immunohistochemistry, immunofluorescence, short tandem repeat analysis, quantitative real-time polymerase chain reaction, and whole-exome sequencing.

**Results:**

A total of 11 subcutaneous xenografts were successfully established from 16 surgical specimens. An increased success rate of implantation in gliomas with wild type isocitrate dehydrogenase (IDH) and high Ki67 expression was observed compared to gliomas with mutant IDH and low Ki67 expression. Recurrent and distant aggressive xenografts were present near the primary implanted tumor fragments from WHO grades II to IV. The xenografts histologically represented the corresponding patient tumor and reconstituted the heterogeneity of different grade gliomas. However, increased Ki67 expression was found in propagated xenografts. Endothelial cells from mice in patient-derived xenografts over several generations replaced the corresponding human tumor blood vessels. Short tandem repeat and whole-exome sequencing analyses indicated that the glioma PDX tumors maintained their genomic features during engraftments over several generations.

**Conclusions:**

The panel of patient-derived glioma xenografts in this study reproduced the diverse heterogeneity of different grade gliomas, thereby allowing the study of the growth characteristics of various glioma types and the identification of tumor-specific molecular markers, which has applications in drug discovery and patient-tailored therapy.

## Background

Gliomas form a heterogeneous group of tumors in the central nervous system (CNS) and are the most common brain tumors [1]. Gliomas, including diffuse gliomas and more circumscribed gliomas, have been classified into grades I to IV by the 2016 World Health Organization (WHO) according to their histology and molecular features [2]. Different grade gliomas have distinct molecular alterations, diverse clinical manifestations, heterogeneity, invasive growth, and resistance to cancer therapies. Thus, there is an urgent need to develop novel therapeutics and companion diagnostics for the molecular and genetic heterogeneous different grade gliomas. For decades, investigations into the pathogenesis underlying glioma and resistance mechanisms have been performed using xenografts based on conventional cancer glioblastoma (GBM) cell lines, such as U87 and U251 [3, 4]. However, these cell line-derived xenografts do not typically reproduce cancer heterogeneity or have therapeutic resistance cues because cultured cell lines suffer from genome and transcriptome alterations caused by in vitro culture conditions over many years [5, 6]. For these reasons, the clinical value of these cell models as an accurate representative tumor model, with some notable exceptions (e.g., U251 GBM cells) [7], has been regularly questioned. It has been increasingly shown that patient-derived xenograft (PDX) models, obtained by engrafting patient tumor fragments or short-term cultured tumor cells from patients into immunodeficient mice, represent a reliable preclinical model for drug development and personalized therapy [8, 9]. Studies on orthotopic GBM xenograft models using primarily cultured GBM cells or GBM stem cells have recapitulated the characteristics of the original tumor [10, 11]. However, only six studies have described PDX models from patient-derived glioma cultures for lower grade glioma because low-grade glioma cells are difficult to grow in vitro and in vivo [12–14]. Moreover, those primarily cultured tumor cell xenograft models are frequently unable to recapitulate the interaction of gliomas with the microenvironment, such as vasculature [15]. Of particular importance, invasiveness and recurrence of subcutaneous PDXs near the site of implantation and in primarily implanted place for gliomas were not observed in any previous publication.

In this study, we established glioma model systems of distinct WHO grades that were derived from patient specimens. Then we analyzed the association between the success rate of implantation with clinical parameters and resemblance to the patient’s original tumor using immunostaining, short tandem repeat (STR) analysis, copy number variation (CNV) and quantitative real-time polymerase chain reaction (qPCR). Here, we established low-grade and high-grade glioma xenografts that reflected the grade-specific characteristics of the original gliomas, allowing preservation of the valuable patient specimens and propagation of the tumor tissue for biobanking and development of glioma intracranial models. This approach may provide novel strategies for drug screening and therapy.

## Methods

### Patients and tumor samples

After obtaining written informed consent, surgical specimens and clinical records were obtained from 16 patients with gliomas who had surgery to remove brain tumors at the First Affiliated Hospital of Chongqing Medical University (Chongqing, China), in accordance with the appropriate Institutional Review Boards (Table 1). At Chongqing Medical University [2], tumors were examined by pathologists and classified based on the 2016 WHO criteria of the CNS. Written informed consent was obtained from all of the patients.

**Table 1:** Clinical characteristics of the patients with gliomas used for implantation

Patient ID number	Age range	Sex	WHO grades	WHO classification	Tumor volume (cm^3^)	IDH1 mutant status	ATRX expression	TP53 expression	Ki67 (%)	1p/19q deletion (%)	Xenograft formation^a^
20161128	51–55	F	II	Diffuse astrocytoma, IDH^WT^	90	WT	NA	NA	30	NA	Y(P3)
20170313	31–35	M	II	OGD, NOS	180	MUT	NA	NA	3–5	NA	N
20170620	56–60	F	II	Astrocytoma, IDH^MUT^	27	MUT	−	+	2	NA	N
20161109	51–55	M	III	Anaplastic OGD, NOS	90	MUT	NA	NA	< 5	NA	N
20170612	71–75	M	III	Anaplastic OGD, NOS	100	WT	+	+	20	29/17	Y(P2)
20170814	61–65	M	III	Anaplastic OGD, IDH^MUT^-1p/19q^c^°^deleted^	288	MUT	+	+	15	53/46	N
20180129	56–60	M	III	Anaplastic OGD, NOS	93.24	WT	+	+	15	73/57	Y(P3)
20180521	56–60	M	III	Anaplastic OGD, IDH^MUT^-1p/19q^c^°^deleted^	180	MUT	+	+	20	27/34	Y(P4)
20161221	66–70	F	IV	GBM, IDH^MUT^	80	MUT	NA	NA	30	NA	N
20170327	56–60	F	IV	GBM, IDH^WT^	180	WT	NA	NA	15	NA	Y(P2)
20170410	56–60	M	IV	GBM (relapse), IDH^WT^	120	WT	NA	+	20	NA	Y(P6)
20170724	61–65	M	IV	GBM, IDH^WT^	78.75	WT	−	+	30	1/5	Y(P2)
20180314	46–50	F	IV	GBM, IDH^WT^	240	WT	+	+	20	5/1	Y(P2)
20180319	66–70	M	IV	GBM, IDH^WT^	180	WT	+	+	30	NA	Y(P5)
20180408	56–60	M	IV	GBM, IDH^WT^	125	WT	+	+	50	NA	Y(P2)
20180515	61–65	F	IV	GBM, IDH^WT^	80	WT	+	−	70	33/45	Y(P2)

WT, wild type; MUT, mutant type; −, negative; +, positive; Y, yes; P, propagation number; N, no; NA, not assessed; OGD, oligodendroglioma; GBM, Glioblastoma; M, male; F, female; IDH, isocitrate dehydrogenase

^a^Whether or not the primary tumor was able to be used to form a xenograft tumor

### Mouse use and care

Specific pathogen-free 6 to 8-week-old female and male NOD-Prkdc^scid^ ll2rg^null^ (NPG) mice were purchased from Beijing Vitalstar Biotechnology Co., Ltd. (Beijing, China), and housed and maintained in the animal facilities of Chongqing Medical University. All animal studies conformed to the Guidelines for the Care and Use of Laboratory Animals.

### Subcutaneous xenograft animal model

Mice were anesthetized with an intraperitoneal injection of 40 mg/mL tribromoethanol (Sigma, St. Louis, MO, USA) diluted in tert-amyl alcohol and normal saline according to a previous report [16]. Specimens from surgery were added to cryopreserved Dulbecco’s Modified Eagle Medium: Nutrient Mixture F12 (Hyclone, Logan, UT, USA) containing 10% fetal calf serum (FCS; Gibco, New York, NY, USA), 1% penicillin, and 1% streptomycin. Tumor specimens were placed in sterile dishes and cut into 3-mm pieces with sterile surgical instruments within 5 h of surgery. A total of two to four tumor pieces were subcutaneously implanted into the right and left flanks of 8 to 12-week-old NPG mice (50% females and 50% males). Implantation was conducted in a laminar flow cabinet using sterilized surgical instruments. Xenografts were measured weekly using vernier calipers in two dimensions when the implanted glioma was tangible. Tumor volume was calculated using the following equation: (width^2^ × length)/2. Latency time, the time until growth was observed, was defined as the time between implantation and the first moment of measurable tumor (~ 13.5 mm^3^) according to the literature [17]. When the tumor volume grew to more than 1500 mm^3^ or animals reached humane endpoints as stated in the Laboratory Animal Guideline for ethical review of animal welfare of Standardization Administration of China (GB/T 35892-2018), the tumor was harvested for propagation or fixed for histological evaluation and snap-frozen in liquid nitrogen for subsequent investigations. The implanted mice were still observed after the surgical removal of primary xenografts to evaluate whether the xenografts preserved the recurrence that characterizes the corresponding patient glioma. We also observed whether distant invasive xenografts were present near the primary implanted tumor fragments to validate the invasiveness of engraftments.

### Histology and immunohistochemistry

Paraffin-embedded tumor tissues from primary and xenograft tumors were sliced into 4 μm sections. Tumor morphology was evaluated by staining with hematoxylin and eosin (H&E) and immunohistochemical (IHC) staining. The slides were deparaffined in xylene, dehydrated in a graded alcohol series, and stained with H&E for histological examination. Heat-mediated epitope retrieval and protease-induced epitope retrieval were used for IHC detection. Then the sections were treated overnight at 4 °C with primary antibodies against glial fibrillary acidic protein (GFAP, anti-human monoclonal antibody, 1:100 dilution, ZA-0529; Zsbio, Beijing, China), vimentin (anti-human monoclonal antibody, 1:100 dilution, ZA-0511; Zsbio, Beijing, China), oligodendrocyte transcription factor 2 (OLIG2, anti-human monoclonal antibody, 1:100 dilution, ZA-0561; Zsbio, Beijing, China), Ki67 (anti-human monoclonal antibody, 1:100 dilution, ZA-0502; Zsbio, Beijing, China). Sections were subsequently incubated with a secondary antibody conjugated with horseradish peroxidase polymer. Next, staining was conducted with DAB substrate, and counterstaining was done with hematoxylin. The ratio of stained positive cells to unstained negative cells was evaluated by counting at least 1000 cells in randomly chosen 400× magnification fields; the scoring the five fields of interest was done by two independent observers in a blind manner.

To determine whether there was replacement of human vasculature by mouse vasculature, tumor cryosections were double-stained with a monoclonal rabbit anti-human cluster of differentiation 31 (CD31) antibody (1:100 dilution, ab180175; Abcam, Cambridge, UK) and a monoclonal rat anti-mouse CD31 antibody (1:100 dilution, 553708; Becton–Dickinson, Franklin Lakes, NJ, USA). Cryosections were subsequently rinsed and labeled simultaneously with FITC-conjugated goat anti-rabbit IgG and Cy3-conjugated goat anti-rat IgG. Lastly, cellular nuclei were counterstained by 40,6-diamidino-2-phenylindole (DAPI, Sigma). At low power field (100×), we chose five fields within the most intensely vascularized fields (so-called ‘‘hot spots’’) under fluorescent microscopy. The vessels of these areas were counted at high power field (400×) by Image Pro- plus 6.0 software and the average microvessel number expressing human CD31 and mouse CD31 in the five fields of interest was scored. Finally, the percentage of anti-human CD31 positive vessels in the total vessels was obtained.

### STR analysis, whole-exome sequencing, and genetic mutation of isocitrate dehydrogenase

Genomic DNA was extracted from representative frozen fragments of each patient’s tumor and their corresponding PDX tumors from the serial passages using a QIAamp DNA Mini Kit (QIAGEN Inc., Hilden, Germany), and it was stored at − 80 °C until analysis. For STR analysis, target DNA was amplified by multiplex PCR for 21 loci using the Microreader™ 21 Direct ID System PCR Amplification Kit (Microread Genetics Co., Ltd, Suzhou, China). PCR products were electrophoresed in the ABI 3130 Genetic Analyzer (Applied Biosystems, Foster City, CA, USA), and analyzed with Gene Mapper ID software (v3.2) using the supplied allelic ladders (Applied Biosystems). For genetic mutation of isocitrate dehydrogenase (IDH) 1 and IDH2, PCR reactions (denaturation at 95 °C for 3–5 min, followed by 35 cycles, starting at 94 °C for 30 s, 58 °C for 30 s, and 72 °C for 30 s) were performed in a 50 µL volume that contained 2 µL DNA, 2 µL each primer, 2 µL Dntp mix, 5 µL 10× Taq Buffer, and 0.5 µL Taq plus DNA (Sangon Biotech, Shanghai, China). PCR products were purified using a SanPrep PCR Purification Kit (Sangon Biotech), and sequencing was performed using the BigDye Terminator Kit (v 1.1; Thermo Fisher Scientific) on the 3730XL genetic analyzer. Whole-exome sequencing was conducted on tumor specimens from five independent patients (20161128, 20170410, 20180129, 20180408, and 20180521) and their matched PDX tumors from different generations. Briefly, the extracted DNA was quantified using a Qubit fluorometer. DNA-seq libraries were generated and captured exome regions using Agilent SureSelect Human All Exon V6. After quality control, DNA libraries were constructed and sequenced to a target depth of 100× for all samples on the Illumina HiSeq platform. After removal of duplicate reads, the base quality control was determined using Cutadapt 0.7.15 software. Of these, variants with low frequency (< 0.05) were first filtered in 1000 genome database and ExAC database, and only the missense variants were maintained to perform final analysis. A single BAM alignment file was saved and used in GATK4 v4.1.0.0 for SNP and indel analysis. The identified mutant locus was annotated by ANNOVAR software. Resulting data were utilized to calculate CNVs across the human reference genome Build 38 (hg38) and were compared among different specimens using CNV kit. PCA was conducted to compare the concordance of mutation results between the tumor and their matched PDXs.

### Statistical analysis

The chi-square test was used to evaluate the association of individual tumor characteristics with engraftment. The percentage of cells that stained positive for Ki-67 was analyzed using the Student’s *t*-test, and data of GFAP, vimentin, and OLIG2 are shown as the mean ± standard deviation. Statistical analysis was conducted using SPSS 17.0 for Windows (SPSS Inc., Chicago, IL, USA). All statistical tests were two-sided, and statistical significance was set at *P *< 0.05.

## Results

### The success rate of glioma xenografts is significantly associated with IDH-wild type and high Ki67 expression in patients

Between November 2016 and May 2018, tumor tissues from 16 patients with gliomas of WHO grades II–IV were implanted in mice. Patient information and tumor histopathology are shown in Table 1. From these, 11 PDX models were successfully established (success rate, 68.8%). The success rates of grades II, III, and IV were 33.33% (1/3), 60.00% (3/5), and 87.50% (7/8), respectively. The characteristics of the original tumors that succeeded or failed to form xenografts were compared retrospectively. The results demonstrated that tumor engraftment was not dependent on patient gender, age, or WHO grade. However, there was a higher frequency of IDH-wild-type gliomas than IDH-mutant gliomas (10/10 vs. 1/6, *P *= 0.001). Moreover, the success rate was significantly associated with high Ki67 expression (> 5%) in the patient tumor samples (11/13 vs. 0/3, *P *=0.004), suggesting that Ki67 activity may impact the success rate.

### Propagated xenografts grow faster than first-generation xenografts

**Fig. 1:**
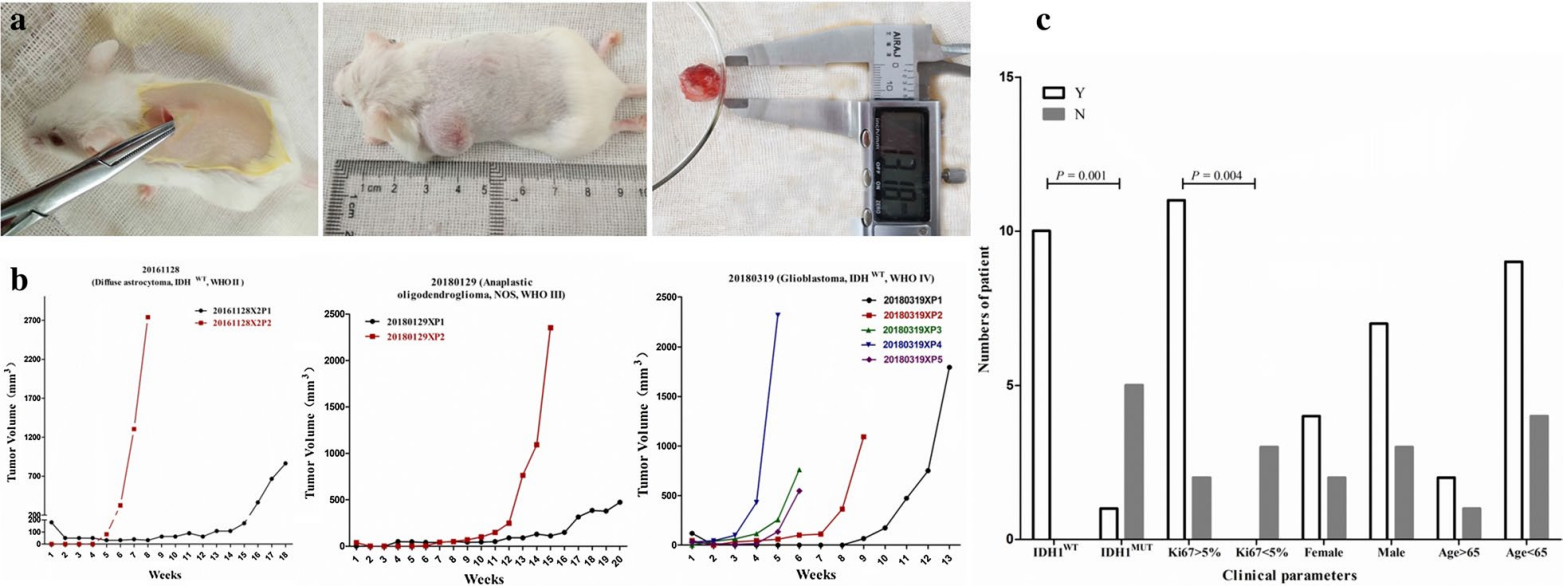
Establishment of the glioma PDX model. **a** Making one to four cuts in the neck or flank, two to four pieces were subcutaneously implanted into the right and left flanks of 8–12 weeks old NPG mice. When the tumor reached the appropriate volume, the tumor was harvested to be propagated, fixed for histological evaluation or snap-frozen in liquid nitrogen for subsequent investigations. **b** Tumor growth of implanted tumor samples from patients 20161128, 20180129 and 20180319 and further propagation of the tumor in successive generations. **c** Tumor success rates were associated with a mutant of IDH and high Ki67 expression (> 5%) in the patient tumor samples

Among the 11 successfully established xenografts, 6 were passaged to second generation, 2 were propagated to the third generation, and a total of 3 were propagated to fourth, fifth, or sixth generations. The median latency time until growth in the first generation was 91 days, but varied from 35 to 154 days among histology subtypes (Table 2). The mean latency period of tumors of WHO grade IV was significantly shorter than that of first-generation tumors of WHO grades II and III. Once the tumors reached a size of around 15 mm or if the mice were in poor health, tumors were harvested and serially transplanted in mice to establish further generations, and were also stored in 95% FCS and 5% DMSO in liquid nitrogen (Fig. 1). Xenograft tumors that were serially passaged showed significantly faster growth compared to first–generation tumors, which suggested that the proliferative capability of passaged xenografts was stronger than that of first–generation tumors, and that PDX models had enhanced proliferative activity of tumor cells.

**Table 2:** Successfully established primary glioma PDXs from November 2016 to May 2018

WHO grades	WHO classification	Cases of established PDXs	PDXs success rate	Latency time (weeks, median range)
II	Diffuse astrocytoma, IDH^WT^	1	1/3	11
III	Anaplastic OGDs, IDH^MUT^-1p/19q^codeleted^	1	60.00% (3/5)	18 (14–22)
	Anaplastic OGDs, NOS	2		
IV	GBM, IDH^WT^	7	87.50% (7/8)	9 (5–22)
Total		11	68.75% (11/16)	13 (5–22)

IDH, isocitrate dehydrogenase; WT, wildtype; MUT, mutant; OGD, oligodendroglioma; GBM, Glioblastoma; PDX, patient-derived xenograft

### Maintenance of invasive and recurrent properties of diffuse gliomas in xenograft tumors

**Fig. 2:**
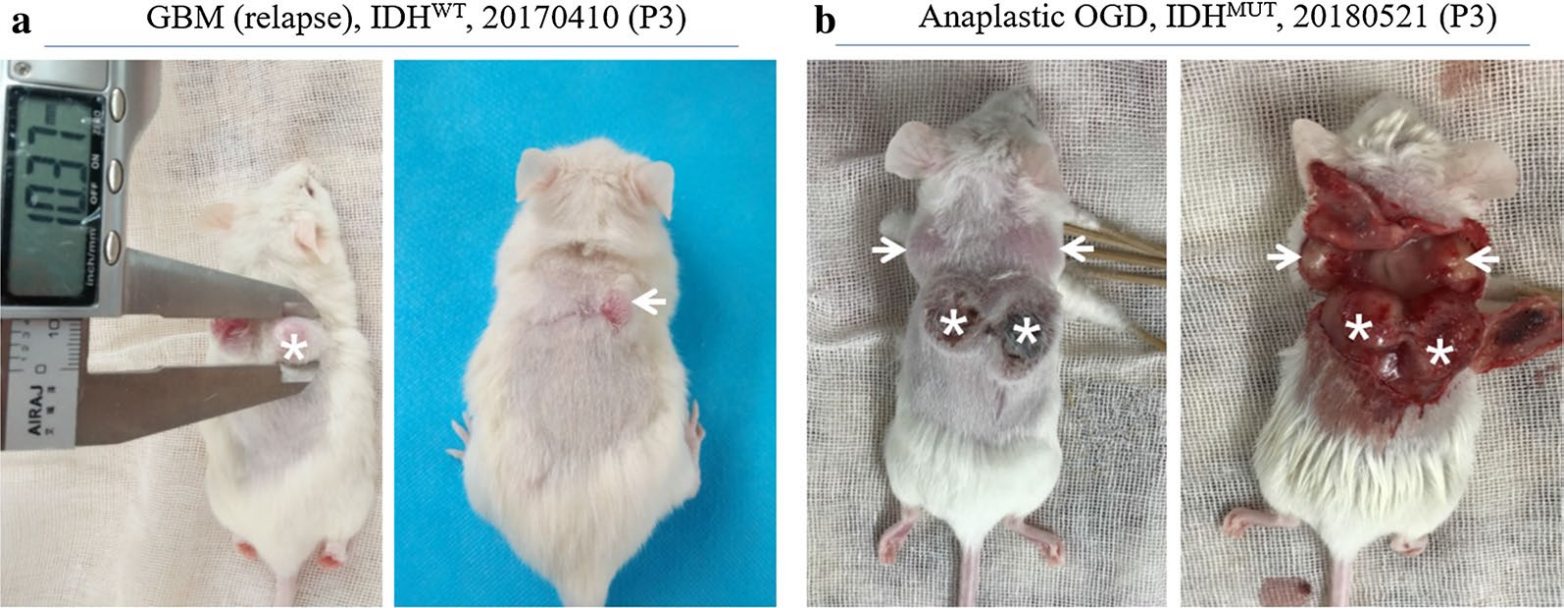
Recurrence and aggressiveness of the glioma xenografts. **a** Recurrence of the glioma xenografts in relapse GBM (IDH^WT^ at its P3 passage). Star shows the primary xenografts. Arrow shows the recurrent xenograft tumor after the removal of primary xenografts. **b** The distant aggressiveness in PDXs (Anaplastic oligodendroglioma, IDH^MUT^ at its P3 passage). Stars show the primary xenografts. Arrows show the distant invasive xenografts from the corresponding xenografts

To investigate the recurrence of xenografts, the xenograft tumors were surgically removed from the tumor-bearing mice and the mice were still observed for evidence of recurrent xenograft tumor growth at the primary implanted location. Interestingly, recurrent xenografts in case 20161128 at its P2 passage and case 20170410 at its P3 passage were observed after the surgical removal of primary xenografts (Fig. 2a). Moreover, distant aggressive xenografts in case 20180521 (Anaplastic Oligodendroglioma, IDH^MUT^) were present near the primary implanted tumor fragments and xenografts at the P1 to P3 passages (Fig. 2b). Recurrent and distant aggressive xenografts were not observed in the other cases.

### Morphology and immunophenotypes of different grade gliomas in xenograft tumors resemble those of patient glioma types

**Fig. 3:**
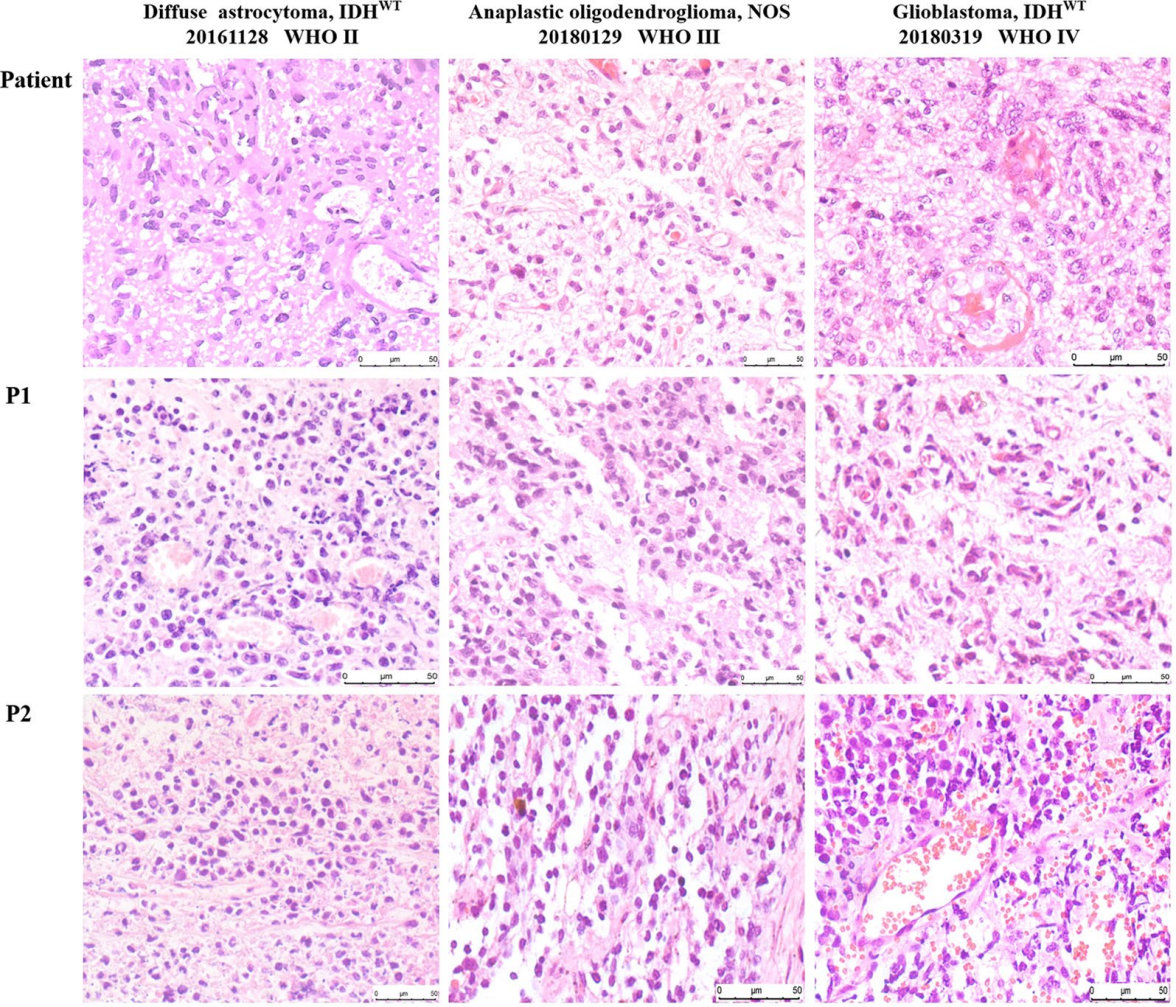
Histology in representative slides of the patient’s primary tumor and xenograft tumor through the generations. The xenografts represented the histological types and preserved the histological features of the corresponding patient tumor

**Fig. 4:**
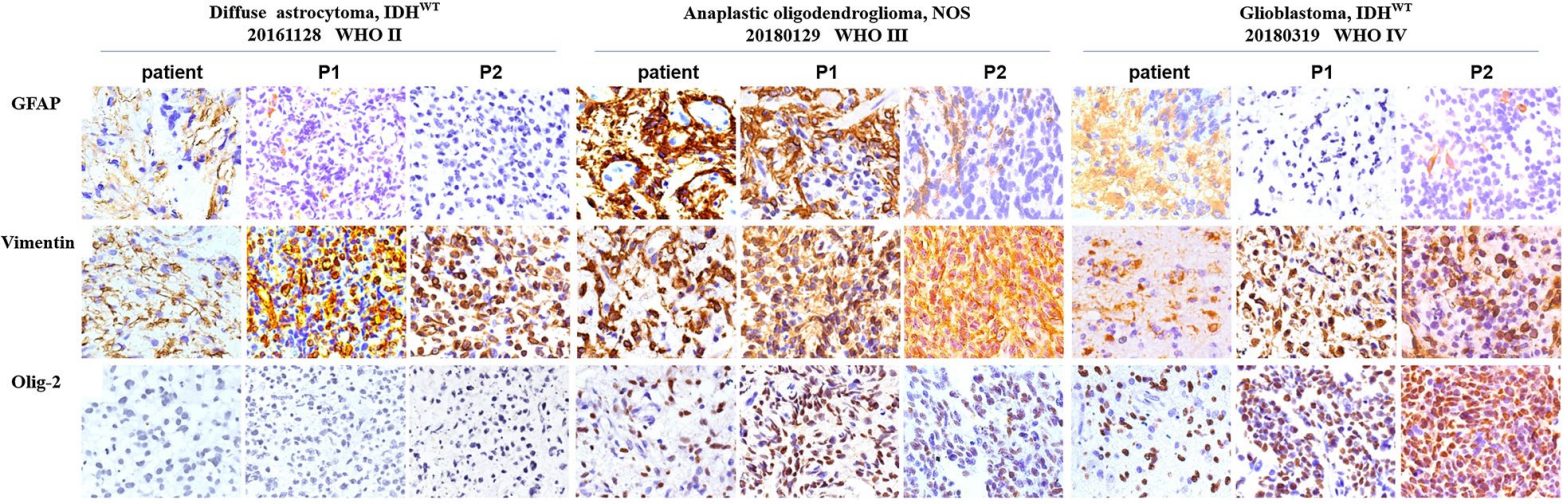
Immunohistochemistry in representative slides of the patient’s primary tumor and xenograft tumor through the generations. The tumor cells of xenografts maintained the key immunophenotype of the patient’s gliomas. There was also decreased GFAP expression and increased vimentin expression over the generations

**Fig. 5:**
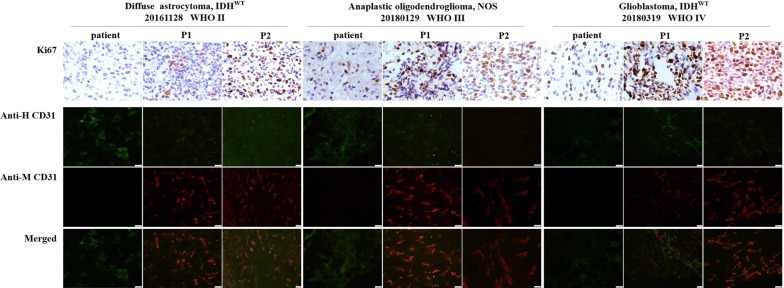
Proliferation activity and vessels of the patient’s primary tumor and xenografts. Proliferation activity using Ki-67 showed high proliferation in all xenografts. Loss of human CD31 and gain of mouse CD31 on endothelial cells lining the vessel walls of xenograft tumor. Magnification ×40 for Ki67, and for CD31 20×

To exclude that the engrafted tumors did not acquire any phenotypic drift, the histology of tumors formed in mice was compared to the matched patient tumor by H&E staining. In all cases, the tissue architecture and morphology of grown xenografts were grossly similar to the corresponding patient tumors (three representative matched cases are shown in Fig. 3). The tumor cells of xenografts maintained the key immune-phenotype of patient gliomas, as determined by staining using GFAP, vimentin and OLIG2. However, there was a trend towards a poorer differentiation with enhanced tumor cell density, nuclear atypia, and endothelial proliferation through increasing generations (Fig. 3). This was also shown by the decreased GFAP expression and increased vimentin expression over generations (Fig. 4 and Additional file 1: Table S1). However, using a monoclonal rat anti-mouse and rabbit anti-human antibody to stain for CD31, increased expression of mouse CD31 on endothelial cells lining the vessel walls in the PDX tumor tissue was observed compared to the primary tumor, suggesting a switch of human to mouse vessels. Proliferative activity, as evaluated by Ki67 staining, showed high proliferation in all propagated xenografts (Fig. 5 and Additional file 2: Figure S1).

### Reproduction of molecular characteristics of human gliomas in glioma xenografts

**Fig. 6:**
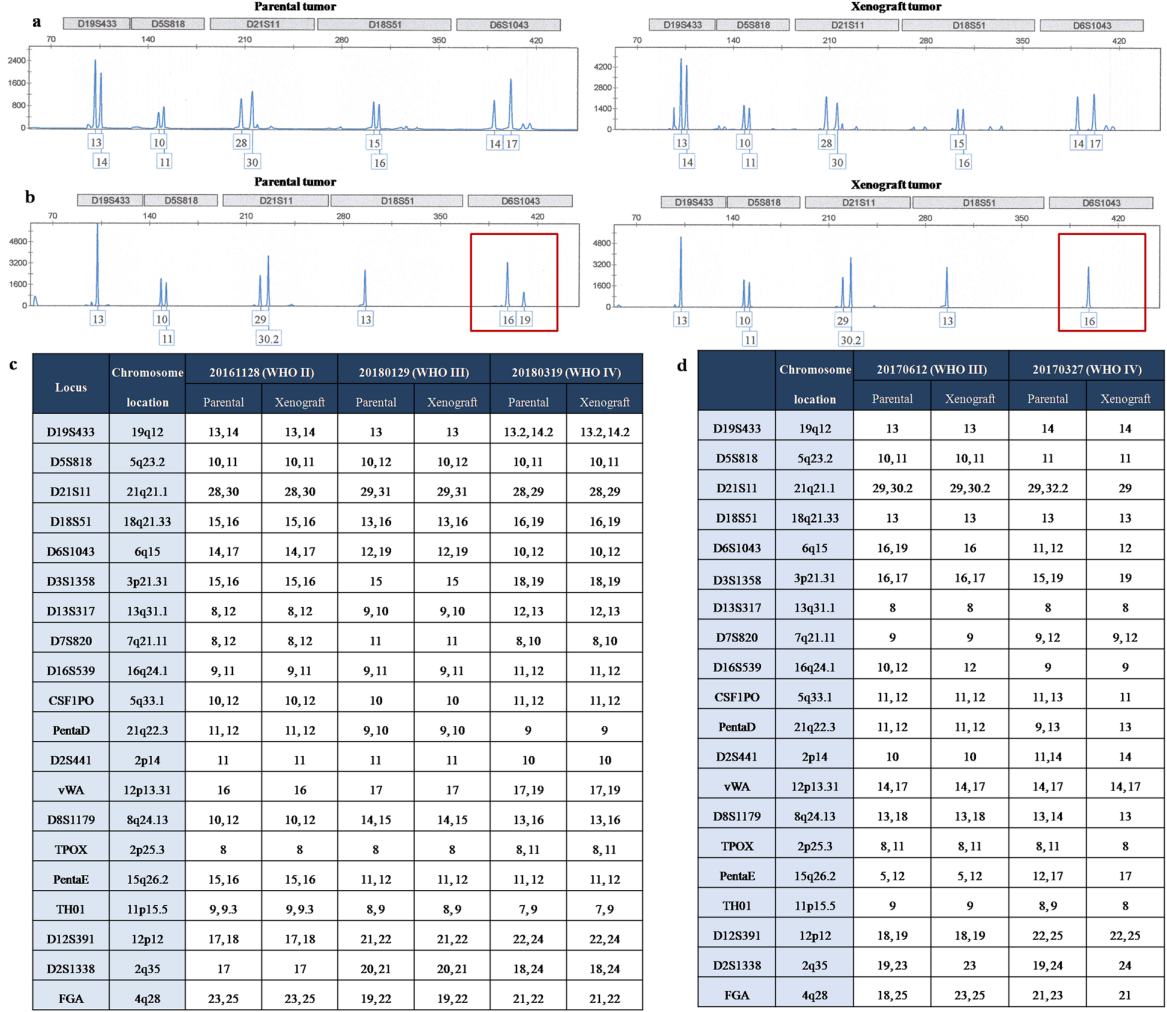
Genetic characteristics of parental and xenograft tumors. **a** Electropherogram showing unique profile of parental tumor and xenograft tumor. **b** Deletion of gene fragments after a few generations. Red box indicates the location of deletion. **c** Genomic consistency of patient and xenograft tumors with short tandem repeat analysis. **d** Genomic variations of patient and xenograft tumors with short tandem repeat analysis

**Fig. 7:**
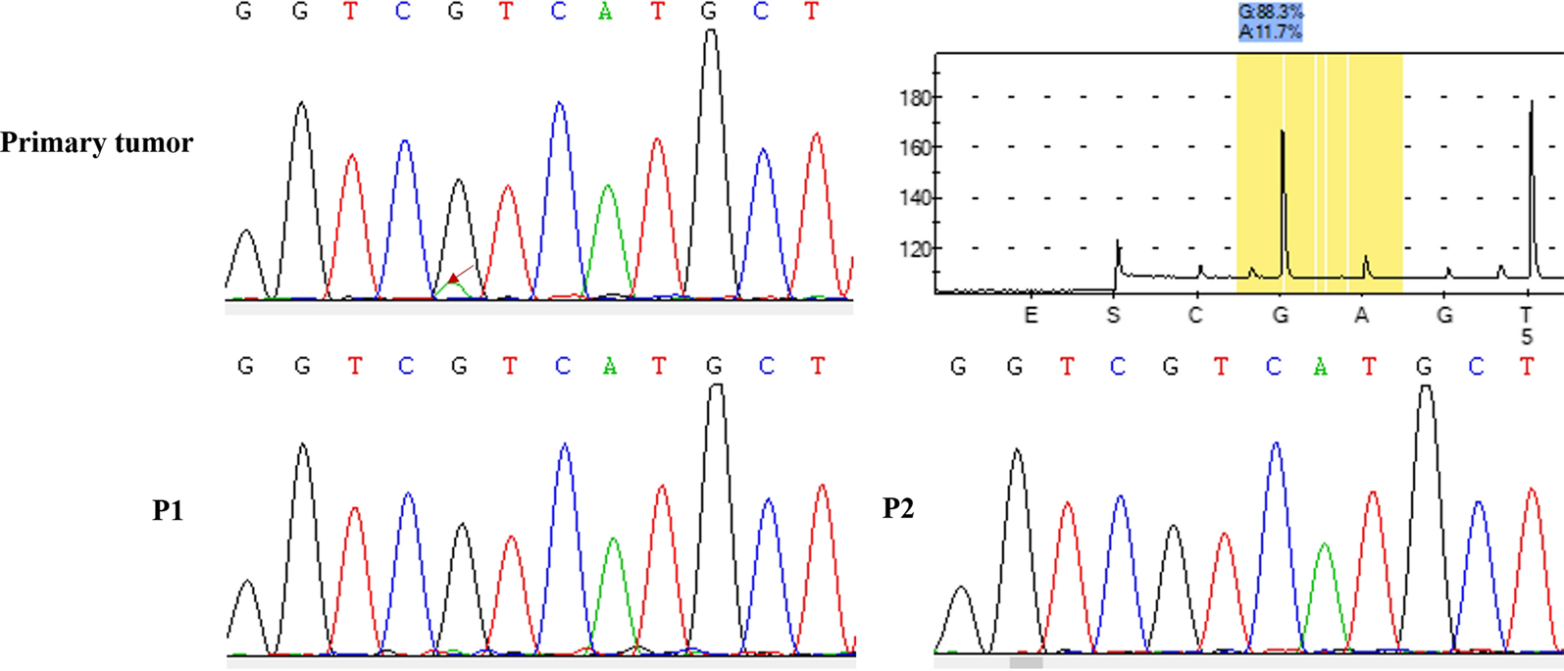
Mutation of IDH in serial passages of the xenograft. Genetic mutations of IDH analysis indicates that 10 cases of the examined genetic mutations through the generations are precisely replicated in the corresponding xenograft tumors, with the exception of case 20180521 in the PDXs losing the R132H mutation. A low heterozygous G/A (11.7%) mutation in the tumor of Patient 20180521. Arrow shows the mutation from G to A

**Fig. 8:**
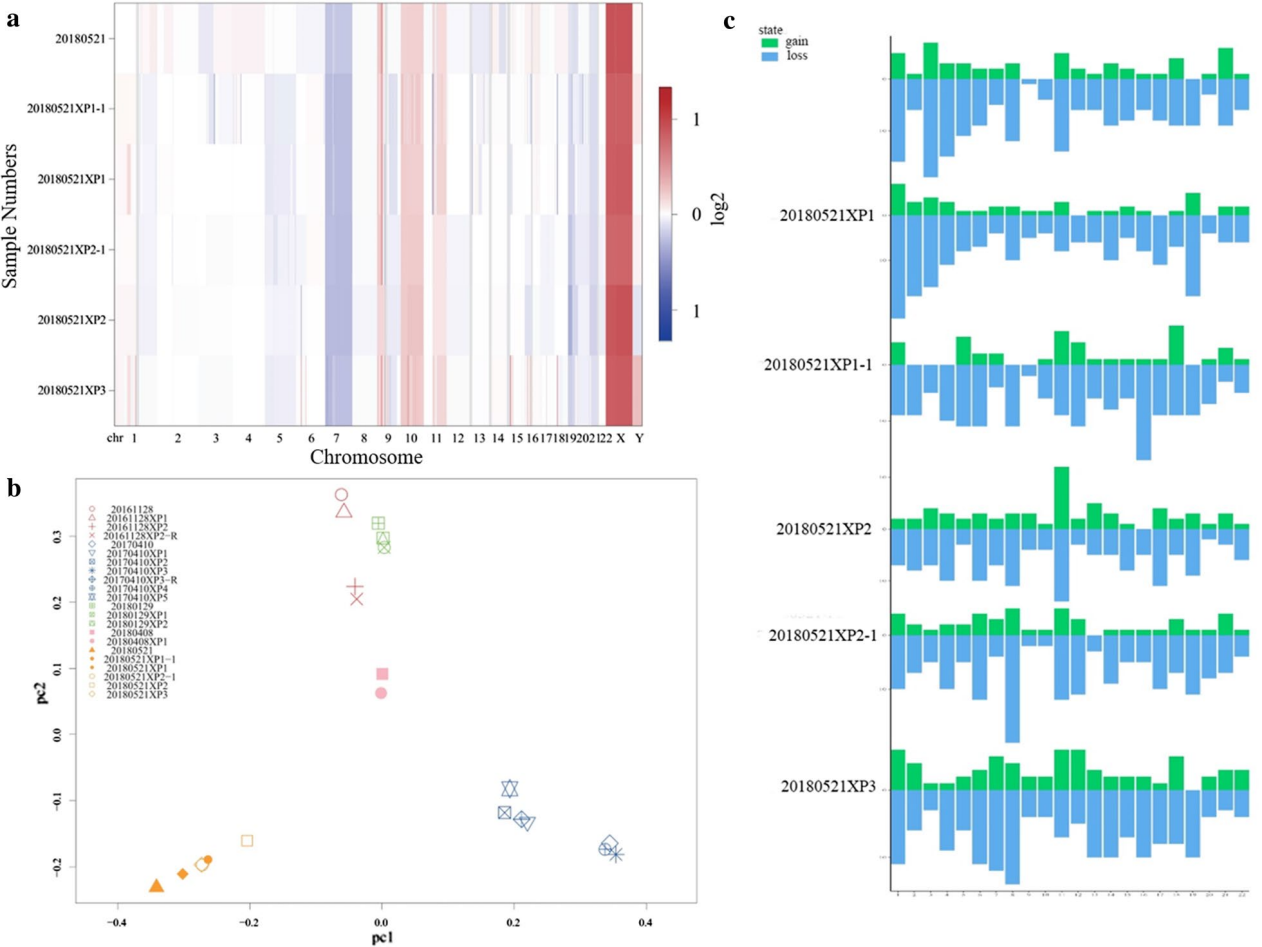
Copy number analysis of parental and their corresponding xenograft tumors using whole-exome sequencing. **a** In the CNV analysis plot, the pattern of CNV of the parental gliomas of patient 20180521 were generally replicated by grafted tumors through the generations and the recurrence and aggressiveness. **b** Principal component analysis (PCA) showed that the tumors from the same patient and their matched PDXs clustered together. **c** Genomic gain is indicated in green and genomic loss is indicated in blue over all chromosomes of the primary tumor of patient 20180521, the corresponding PDXs (P1 to P3) and the distance growth tumors

To further evaluate the similarities between xenograft tumors through the generations and the corresponding parental tumors, we conducted a genomic analysis. First, STR genotyping was performed to ensure that each glioma xenograft was derived from the matched patient with glioma (Fig. 6). Significant heterogeneity was observed among tumors from different patients. In general, the genomic consistency in the majority of tumors from the same patient and their corresponding PDX models was rigorously maintained (Fig. 6a, c), with the exception of some alterations that were observed in cases 20170612 and 20170327 (Fig. 6b, d). To confirm the diagnosis, we PCR-amplified IDH1 and IDH2. Analysis of the genetic mutations of IDH indicated that 10 cases of the examined genetic mutations through generated or recurrent xenografts precisely replicated in the corresponding xenograft tumors, with the exception of case 20180521, revealed a low heterozygous G/A (11.7%) mutation in the original patient tumor that altered to a GG genotype in the xenografts through serial generations and distant aggressive tumors (Fig. 7). In whole-exome sequencing, all the samples were passed the QC criteria including average rate of quality value 30 (Q30) > 80% and average error rate < 0.1%. CNV analysis showed that the CNV pattern the parental patient gliomas was generally replicated by grafted tumors through generation, recurrence, and aggressiveness. PCA showed that there was significant heterogeneity among different patient’s tumors. However, the tumors from the same patient and their matched PDXs clustered together (Fig. 8).

## Discussion

In this study, we established a panel of 11 glioma PDX models from 16 fresh tissues of different grade gliomas according to 2016 CNS WHO grading, which reflected all major histological and genetic characteristics of different grade gliomas. Previous studies have shown that the success rate of brain tumor PDX models varies between 80 and 90% [11]. However, it has also been reported that implantation of brain tumor tissue fragments has failed [18]. In accordance with previous reports, we had a total success rate of 68.75% that varied from 33.33 to 87.50% depending on the different WHO grades by subcutaneous PDX. A tendency for an increased success rate was shown for grades IV and grade III in contrast to grade II, although there was no significant increase in the success rate among the three grades. In our experience, the WHO grade is one of the key factors that determine success rate because the grades are directly associated with the malignant degree. Therefore, it is plausible that the success rate is high for grade IV, and gradually decreases for grades III and II. From the IDH status perspective, IDH-wild type gliomas significantly increased the success rate compared to IDH-mutant gliomas, possibly because the IDH mutation is an early driver of gliomagenesis [19]. Ki67 is directly associated with proliferative ability; the expression of Ki67 significantly enhanced the success rate of the engrafts by more than 5%. This result is similar to the findings of previous studies in lung cancer and ovarian tumor PDX models [17, 20]. Therefore, these results suggest that successful engraftment of tumor tissue is dependent on both tumor-type and Ki67 expression.

Invasive growth and tumor recurrence are the two key characteristics of diffuse gliomas in humans. Invasive patient-derived orthotopic xenografts (PDOXs) for gliomas have been modeled from patient surgical specimens and patient-derived short-term cultures [11, 21, 22]. In addition, invasive orthotopic gliomas have been established from subcutaneous xenografts from surgical specimens of patients with gliomas and from their propagations [23]. However, there is no report of invasive growth and tumor recurrence in glioma subcutaneous PDX models. In the present study, recurrence of xenografts in diffuse astrocytoma at its P2 passage and in the recurrent GBM at its P3 passage was observed after the surgical removal of primary xenografts. Moreover, a spreading of isolated xenografts into the adjacent connective tissue was observed in the primary implanted tumor fragments and xenografts at the P1 to P3 passages in anaplastic oligodendroglioma. These results suggest that the PDX models in our study recapitulated the invasive properties of human malignant gliomas.

We further characterized the histopathology of the parental patient and xenograft tumors. We showed that xenograft tumors maintained the major histologic and key immunophenotypic features of the original tumor, with the exception of differentiation degree, which showed decreased GFAP expression in the propagated xenograft tumors compared with the corresponding original patient tumors. Additionally, the gain of mouse CD31 suggests replacement of vascularization by the murine host, as previously reported in an ovarian cancer PDX model [17]. Moreover, variations in the expression of Ki67 were observed. This result may partially explain why the growth of xenograft tumors in mice was faster in serial passages. To further confirm the classification, we detected IDH status. There were no differences in IDH1/IDH2 compared with original tumors, except that patient tumor of the case 20180521 harbored a low mutation of IDH1 (R132H), but the xenografts altered into an IDH-wildtype tumor through the serial passages and recurrences. We reasoned that a low level of IDH1 (11.7%) mutation in the patient tumor may easily loss in the later serial transplantation and account for the alteration of IDH status in xenografts. In conclusion, the engrafted PDX tumors retained the morphologic and molecular characteristics of the original tumors.

Previous studies have demonstrated that there is a high concordance of genomic variation between primary tumors and corresponding xenograft tumors and therefore PDX models currently represent the most reliable models in tumor biological research and preclinical studies [11, 24, 25]. However, genomic variation in PDX models compared with parental patient tumors has also been demonstrated [26, 27]. The main factors contributing to these genomic alterations can be explained by the enrichment of human tumor DNA after the loss of human stromal cells during propagation in mice. A recent biobanking study of patient-derived ovarian cancer (PDX tumors) using genome-wide single nucleotide polymorphism microarray analysis, showed high concordance with the original tumors [17]. In alignment with previous results, we found that prominent genetic patterns of patient tumor were typically maintained in the engrafted tumors, although there were deletions in two cases after a few generations, which ultimately recovered the original genetic characteristics. The STR results and CNV analysis supported our confirmed molecular mutant data for PDX tumors.

In conclusion, the results of the current study showed that we successfully established a panel of various glioma PDX models that reflected the different histopathologic and genetic characteristics of the original gliomas.

## Supplementary information

**Additional file 1: Table S1.** Semi-quantitative analysis of immunehistochemistry of primary tumors and xenograft tumors.

**Additional file 2: Figure S1.** Proliferation activity and vessels of the patient’s primary tumor and xenografts. Proliferation index was analyzed by immunohistochemistry against anti-human Ki-67 and then calculated the positive cell rate of patient gliomas and corresponding xenografts. Compared with patients, a significant increase was observed in xenografts (*P *= 0.035).

## Data Availability

All data generated or analyzed during this study are included in this published article.

## Abbreviations

IDH: isocitrate dehydrogenase
GFAP: glial fibrillary acidic protein
OLIG2: oligodendrocyte transcription factor 2
CNS: central nervous system
WHO: World Health Organization
GBM: glioblastoma
PDX: patient-derived xenograft
PDOX: patient-derived orthotopic xenograft
STR: short tandem repeat analysis
qPCR: quantitative real-time polymerase chain reaction
NPG mice: NOD-Prkdc^scid^ ll2rg^null^ mice
FCS: fetal calf serum
DMSO: dimethyl sulfoxide
H&E: hematoxylin and eosin
CD31: cluster of differentiation 31

## References

[1] Ostrom QT, Gittleman H, Farah P, Ondracek A, Chen Y, Wolinsky Y, Stroup NE, Kruchko C, Barnholtz-Sloan JS (2013). CBTRUS statistical report: primary brain and central nervous system tumors diagnosed in the United States in 2006–2010. Neuro Oncol..

[2] Louis DN, Perry A, Reifenberger G, von Deimling A, Figarella-Branger D, Cavenee WK, Ohgaki H, Wiestler OD, Kleihues P, Ellison DW (2016). The 2016 World Health Organization classification of tumors of the central nervous system: a summary. Acta Neuropathol.

[3] Schmidt NO, Ziu M, Carrabba G, Giussani C, Bello L, Sun Y, Schmidt K, Albert M, Black PM, Carroll RS (2004). Antiangiogenic therapy by local intracerebral microinfusion improves treatment efficiency and survival in an orthotopic human glioblastoma model. Clin Cancer Res.

[4] Aghi M, Rabkin S, Martuza RL (2006). Effect of chemotherapy-induced DNA repair on oncolytic herpes simplex viral replication. J Natl Cancer Inst.

[5] Gillet JP, Varma S, Gottesman MM (2013). The clinical relevance of cancer cell lines. J Natl Cancer Inst.

[6] Allen M, Bjerke M, Edlund H, Nelander S, Westermark B (2016). Origin of the U87MG glioma cell line: good news and bad news. Sci Transl Med..

[7] Radaelli E, Ceruti R, Patton V, Russo M, Degrassi A, Croci V, Caprera F, Stortini G, Scanziani E, Pesenti E (2009). Immunohistopathological and neuroimaging characterization of murine orthotopic xenograft models of glioblastoma multiforme recapitulating the most salient features of human disease. Histol Histopathol.

[8] Patrizii M, Bartucci M, Pine SR, Sabaawy HE (2018). Utility of glioblastoma patient-derived orthotopic xenografts in drug discovery and personalized therapy. Front Oncol.

[9] Pine SR, Sabaawy HE (2018). Editorial: harnessing the power of patient derived models of cancer. Front Oncol.

[10] Irtenkauf SM, Sobiechowski S, Hasselbach LA, Nelson KK, Transou AD, Carlton ET, Mikkelsen T, deCarvalho AC (2017). Optimization of glioblastoma mouse orthotopic xenograft models for translational research. Comp Med.

[11] Joo KM, Kim J, Jin J, Kim M, Seol HJ, Muradov J, Yang H, Choi YL, Park WY, Kong DS (2013). Patient-specific orthotopic glioblastoma xenograft models recapitulate the histopathology and biology of human glioblastomas in situ. Cell Rep.

[12] Le Mercier M, Fortin S, Mathieu V, Roland I, Spiegl-Kreinecker S, Haibe-Kains B, Bontempi G, Decaestecker C, Berger W, Lefranc F (2009). Galectin 1 proangiogenic and promigratory effects in the Hs683 oligodendroglioma model are partly mediated through the control of BEX2 expression. Neoplasia.

[13] Klink B, Miletic H, Stieber D, Huszthy PC, Campos Valenzuela JA, Balss J, Wang J, Schubert M, Sakariassen PO, Sundstrom T (2013). A novel, diffusely infiltrative xenograft model of human anaplastic oligodendroglioma with mutations in FUBP1, CIC, and IDH1. PLoS ONE.

[14] Navis AC, Niclou SP, Fack F, Stieber D, van Lith S, Verrijp K, Wright A, Stauber J, Tops B, Otte-Holler I (2013). Increased mitochondrial activity in a novel IDH1-R132H mutant human oligodendroglioma xenograft model: in situ detection of 2-HG and alpha-KG. Acta Neuropathol Commun.

[15] Carrillo MA, Zhen AJ, Kitchen SG (2018). The use of the humanized mouse model in gene therapy and immunotherapy for HIV and cancer. Front Immunol..

[16] Li L, Hua ZD, Ren HY, Bi YZ, Liu XM, Xiao HW, Zhang LP, Zheng XM (2015). Anesthetic effects of tribromoethanol on mice. Hubei Agr Sci.

[17] Alkema NG, Tomar T, Duiker EW, Jan Meersma G, Klip H, van der Zee AG, Wisman GB, de Jong S (2015). Biobanking of patient and patient-derived xenograft ovarian tumour tissue: efficient preservation with low and high fetal calf serum based methods. Sci Rep.

[18] Kim KM, Shim JK, Chang JH, Lee JH, Kim SH, Choi J, Park J, Kim EH, Kim SH, Huh YM (2016). Failure of a patient-derived xenograft for brain tumor model prepared by implantation of tissue fragments. Cancer Cell Int..

[19] Picca A, Berzero G, Di Stefano AL, Sanson M (2018). The clinical use of IDH1 and IDH2 mutations in gliomas. Expert Rev Mol Diagn.

[20] Lu D, Luo P, Zhang J, Ye Y, Wang Q, Li M, Zhou H, Xie M, Wang B (2018). Patient-derived tumor xenografts of lung squamous cell carcinoma alter long non-coding RNA profile but not responsiveness to cisplatin. Oncol Lett.

[21] Fei XF, Zhang QB, Dong J, Diao Y, Wang ZM, Li RJ, Wu ZC, Wang AD, Lan Q, Zhang SM (2010). Development of clinically relevant orthotopic xenograft mouse model of metastatic lung cancer and glioblastoma through surgical tumor tissues injection with trocar. J Exp Clin Cancer Res.

[22] Larsson S, Wenger A, Dosa S, Sabel M, Kling T, Caren H (2018). Cell line-based xenograft mouse model of paediatric glioma stem cells mirrors the clinical course of the patient. Carcinogenesis.

[23] Taillandier L, Antunes L, Angioi-Duprez KS (2003). Models for neuro-oncological preclinical studies: solid orthotopic and heterotopic grafts of human gliomas into nude mice. J Neurosci Methods.

[24] Hidalgo M, Amant F, Biankin AV, Budinska E, Byrne AT, Caldas C, Clarke RB, de Jong S, Jonkers J, Maelandsmo GM (2014). Patient-derived xenograft models: an emerging platform for translational cancer research. Cancer Discov.

[25] da Hora CC, Schweiger MW, Wurdinger T, Tannous BA (2019). Patient-derived glioma models: from patients to dish to animals. Cells..

[26] DeRose YS, Wang G, Lin YC, Bernard PS, Buys SS, Ebbert MT, Factor R, Matsen C, Milash BA, Nelson E (2011). Tumor grafts derived from women with breast cancer authentically reflect tumor pathology, growth, metastasis and disease outcomes. Nat Med.

[27] Press JZ, Kenyon JA, Xue H, Miller MA, De Luca A, Miller DM, Huntsman DG, Gilks CB, McAlpine JN, Wang YZ (2008). Xenografts of primary human gynecological tumors grown under the renal capsule of NOD/SCID mice show genetic stability during serial transplantation and respond to cytotoxic chemotherapy. Gynecol Oncol.

