# Supplementary material for: Patient-derived xenografts of different grade gliomas retain the heterogeneous histological and genetic features of human gliomas
Source: Cancer Cell Int. 2020 Jan 3;20:1. doi: 10.1186/s12935-019-1086-5 (PMC6941273; doi:10.1186/s12935-019-1086-5)
Supplement: Supplementary file 1 — Additional file 1: Table S1. Semi-quantitative analysis of immunehistochemistry of primary tumors and xenograft tumors. [file 12935_2019_1086_MOESM1_ESM.pdf]

	GFAP(mean density)<br>mean ± standard deviation	Vimentin(mean density)<br>mean ± standard deviation	Olig-2(positive cell rate)<br>mean ± standard deviation
20161128	0.28±0.04	0.5±0.05	0
20161128XP1	0.46±0.16	0.74±0.08	0
20161128XP2	0.32±0.05	0.47±0.05	0
20180129	0.42±0.05	0.55±0.06	50%±0.07
20180129XP1	0.41±0.03	0.39±0.05	85%±0.07
20180129XP2	0.27±0.01	0.24±0.01	53%±0.05
20180319	0.38±0.07	0.37±0.06	58%±0.09
20180319XP1	0	0.48±0.05	69%±0.09
20180319XP2	0.24±0.03	0.29±0.06	72%±0.12
