# Supplementary figures and images for: Patient-derived xenografts of different grade gliomas retain the heterogeneous histological and genetic features of human gliomas
Source: Cancer Cell Int. 2020 Jan 3;20:1. doi: 10.1186/s12935-019-1086-5 (PMC6941273; doi:10.1186/s12935-019-1086-5)

# Ki67 proliferation index

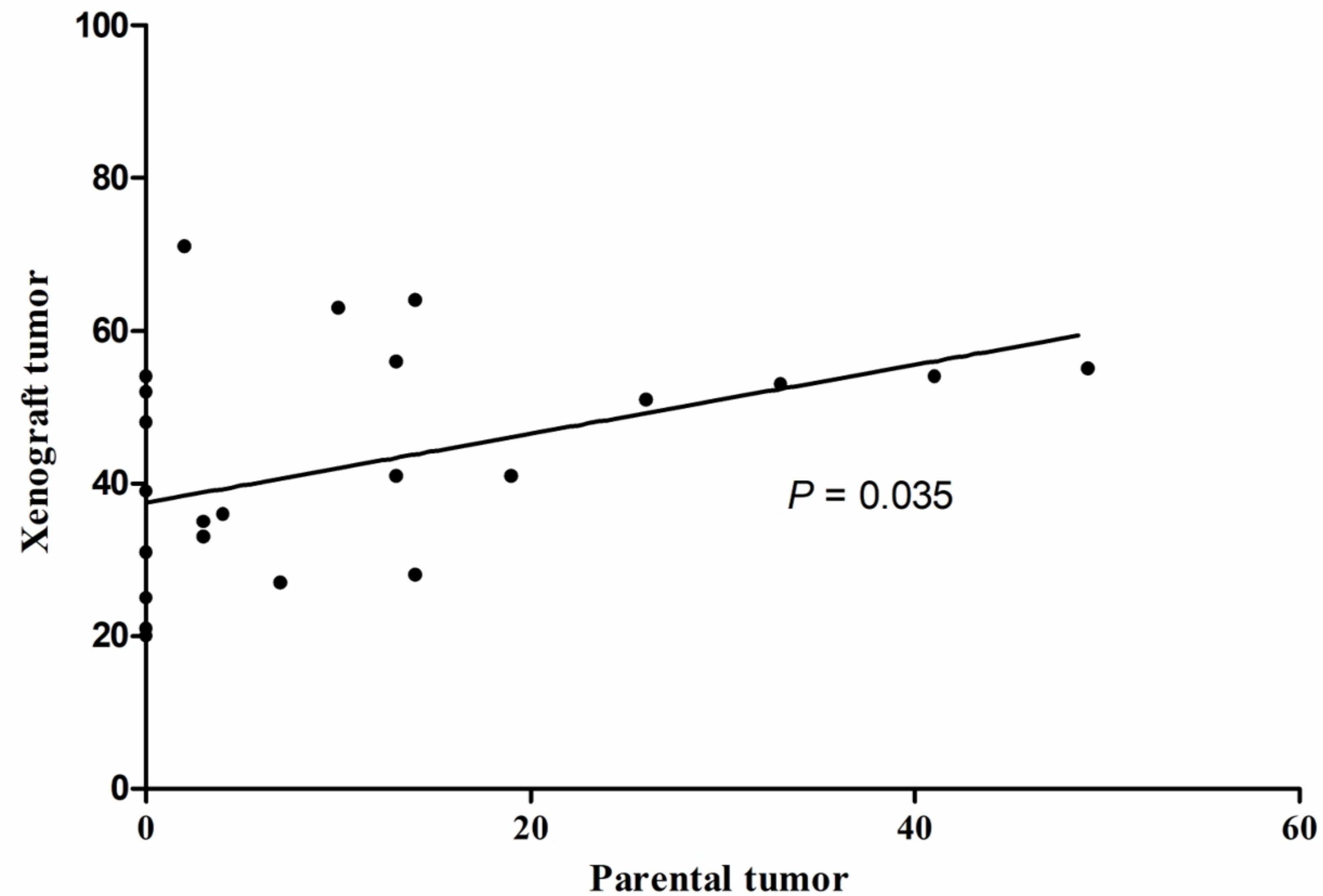

Supplement: Supplementary file 2 — Additional file 2: Figure S1. Proliferation activity and vessels of the patient’s primary tumor and xenografts. Proliferation index was analyzed by immunohistochemistry against anti-human Ki-67 and then calculated the positive cell rate of patient gliomas and corresponding xenografts. Compared with patients, a significant increase was observed in xenografts (P = 0.035). [file 12935_2019_1086_MOESM2_ESM.pdf]
